# Tobacco-Derived Lipopolysaccharide, Not Microbial Translocation, as a Potential Contributor to the Pathogenesis of Rheumatoid Arthritis

**DOI:** 10.1155/2019/4693870

**Published:** 2019-11-03

**Authors:** Pieter W. A. Meyer, Mahmood M. T. M. Ally, Mohammed Tikly, Gregory Tintinger, Lai Ling Winchow, Helen Steel, Ronald Anderson

**Affiliations:** ^1^Department of Immunology, Tshwane Academic Division, National Health Laboratory Services, Pretoria 0001, South Africa; ^2^Department of Immunology, Faculty of Health Sciences, University of Pretoria, Pretoria 0001, South Africa; ^3^Department of Internal Medicine, Faculty of Health Sciences, University of Pretoria, Pretoria 0001, South Africa; ^4^Division of Rheumatology, Chris Hani Baragwaneth Academic Hospital, Faculty of Health Sciences, University of the Witwatersrand, Chris Hani Road, Johannesburg 2013, South Africa

## Abstract

Microbial lipopolysaccharides (LPS) have been implicated in the pathogenesis of rheumatoid arthritis (RA), possibly driving a systemic inflammatory response that may trigger the development and/or exacerbation of the disease. To explore the existence of this mechanism in African RA patients, we have measured systemic levels of LPS and its surrogate, LPS-binding protein (LBP), as well as those of intestinal fatty acid-binding protein (I-FABP), pulmonary surfactant protein D (SP-D), and cotinine in serum to identify possible origins of LPS, as well as associations of these biomarkers with rheumatoid factor (RF) and anticitrullinated peptide (aCCP) autoantibodies and the DAS 28-3 clinical disease severity score. A cohort of 40 disease-modifying antirheumatic drug-naïve, black South African RA patients rated by compound disease scores and 20 healthy subjects and 10 patients with chronic obstructive pulmonary disease (COPD) as controls were included in this study. Levels of the various biomarkers and autoantibodies were measured using a combination of ELISA and immunofluorimetric and immunoturbidometric procedures. LPS levels were lowest in the RA group compared to the healthy controls (*p* = 0.026) and COPD patients (*p* = 0.017), while LBP levels were also significantly lower in RA compared to the healthy individuals (*p* = 0.036). Levels of I-FABP and SP-D were comparable between all three groups. Categorisation of RA patients according to tobacco usage revealed the following significant positive correlations: LBP with C-reactive protein (*p* = 0.0137); a trend (*p* = 0.073) towards an association of LBP with the DAS 28-3 disease severity score; RF-IgG antibodies with both LPS and LBP (*p* = 0.033 and *p* = 0.041, respectively); aCCP-IgG antibodies with LPS (*p* = 0.044); and aCCP-IgG with RF-IgM autoantibodies (*p* = 0.0016). The findings of this study, several of them novel, imply that tobacco products, as opposed to microbial translocation, represent a potential source of LPS in this study cohort of RA patients, again underscoring the risks posed by tobacco usage for the development and severity of RA.

## 1. Introduction

Evidence from earlier studies, both direct [1–5] and indirect [6–8], has implicated enteric bacteria and their proinflammatory products in the pathogenesis of rheumatoid arthritis (RA). However, it is only fairly recently that this concept has been revisited following the acquisition of insights into the role of the composition of the gut microbiome, as well as the structural integrity of the gut mucosa, in orchestrating immune responses [9–12]. This latter scenario involves leakage of bacterial products, especially lipopolysaccharides (LPS) and nucleic acids, from the gastrointestinal tract (GIT) and possibly from other anatomical sites, a process known as microbial translocation. These bacterial products, in turn, may trigger systemic, as well as localised, chronic inflammatory processes, especially in the lung and synovium, that may be linked to the development and perpetuation of RA [8]. In this context, it is noteworthy that bacterial LPS is known to initiate inflammatory mechanisms that cause protein citrullination [13], an event intimately linked to the immunopathogenesis of RA. In this context, autoantibodies to citrullinated proteins/peptides are not only diagnostic for RA but are also indicative of severe erosive disease [14–16]. Importantly, bacterial products are also present in cured tobacco [17–19], with smoking now well recognised as being a major risk factor for RA [20].

However, little is known about the possible involvement of microbial products in activating inflammatory mechanisms that may contribute to the pathogenesis of RA, particularly in the setting of the indigenous peoples of sub-Saharan Africa, who appear to experience a more severe form of disease [21–25]. To explore this issue in black South African RA patients, systemic concentrations of bacterial LPS and LPS-binding protein (LBP) were measured as indicators of endotoxaemia, while surfactant protein D (SP-D) and intestinal fatty acid-binding protein (I-FABP) were included as possible biomarkers of leakage from the lungs and GIT, respectively. LBP is a type 1 acute phase protein, similar to CRP and serum amyloid A (SAA), that recognises and binds the lipid A component of microbial LPS and is indicative of the host-defense response to proinflammatory endotoxins; SP-D is a collectin (a family of microbial pattern recognition receptors with opsonic properties) most commonly, but not exclusively, localised to the lungs, while I-FABP is expressed intracellularly in epithelial cells of the mucosa and is a systemic biomarker of epithelial leakage. Systemic concentrations of these various biomarkers of microbial translocation were then correlated with clinical and serological indices of disease activity.

## 2. Materials and Methods

The study cohort consisted of forty black South African RA patients recruited in 2013 from the rheumatology clinics of two tertiary academic hospitals in the Gauteng Province of South Africa. The patients complied with the 2010 ACR/EULAR RA criteria [26] and were disease-modifying antirheumatic drug- (DMARD-) naïve at baseline. Aliquots of serum prepared from coagulated blood were stored at -80°C until measurement of the various test analytes described below. The control group consisted of 20 anonymous, matched, healthy blood donors (approval: 2017/18, South African National Blood Transfusion Service). To identify possible effects of chronic inflammatory lung disease on the test biomarkers, most importantly the utility of measurement of SP-D as a biomarker of pulmonary damage and leakage, an additional control group of 10 patients (5 female, 5 male) diagnosed with chronic obstructive pulmonary disease (COPD) was recruited from the Lung Unit, Department of Internal Medicine, Steve Biko Academic Hospital, Pretoria. They were all current or ex-tobacco users, and COPD was diagnosed by means of pulmonary function testing according to a FEV_1_/FVC ratio of <70%. The mean postbronchodilator FEV_1_ was 69% of predicted, ranging from 36% of predicted (GOLD Stage III) to 102% of predicted (GOLD Stage I).

The study was approved by the Research Ethics Committee of the Faculty of Health Sciences of the University Pretoria, and informed, signed consent was obtained from all participants as required (approval number: 320/2016).

A chromogenic end-point limulus amoebocyte lysate method (LPS) (QCL-1000™, Lonza Bioscience, Basel, Switzerland USA) was used to quantitate bacterial LPS with the results expressed as enzyme units (EU)/ml serum, while LBP, SP-D, and I-FABP were assayed using ELISA procedures (all from Hycult Biotech, Netherlands) with the results expressed as nanograms (ng)/ml, micrograms (*μ*g)/ml, and picograms (pg)/ml serum, respectively. Serum cotinine levels were measured using an ELISA procedure (Calbiotech, Spring Valley, CA, USA) to verify the exposure to tobacco products in all groups, with results expressed as ng/ml serum and a cut-off of >5 ng/ml to identify those exposed to cigarettes, snuff, and other related tobacco products [27]. Anticitrullinated cyclic peptide (aCCP) IgG/IgA autoantibodies and rheumatoid factor (RF) IgG/IgA/IgM were determined using fluorimetric enzyme immunoassay procedures (EliA, Phadia AB, Oslo, Norway) with results expressed as autoantibody units (U)/ml serum, while high-sensitivity C-reactive protein (hs-CRP) was measured by laser nephelometry (Siemens AG, Munich, Germany) with results expressed as *μ*g/ml serum.

All RA patients were assessed clinically using several validated clinical disease scores (CDAI, SDAI, and DAS 28) [28, 29].

### 2.1. Statistical Analysis

Descriptive and inferential statistical procedures were used in the analysis of data. Tests for the association of contingency tables were performed using two-tailed chi^2^ tests. One-way ANOVA was performed using the Kruskal-Wallis test for nonparametric data for more than 2 groups or Dunn's test of multiple comparisons using rank sums. The Mann-Whitney test was applied when 2 groups were compared or the Wilcoxon test when the equality of matched pairs was determined. Correlation coefficients were derived from correlation matrices using the nonparametric Spearman's rank correlation test for multiple testing. The Bonferroni-Holm's correction was applied in the case of multiple correlations. Statistical significance was determined by a *p* value < 0.05 with statistical trending set at *p* ≤ 0.1 to identify possible underlying significance. The analyses were done using Stata Statistical Software: Release 15 (StataCorp LLC, College Station, TX).

## 3. Results and Discussion

Demographic and duration of disease data, disease activity scores, and serum concentrations of hs-CRP, as well as those of anti-CCP and RF autoantibodies for the group of RA patients are shown in Table 1. The median age and gender ratio (F : M) of the group of COPD control patients were 67 years and 1 : 1, respectively. Serum cotinine levels of the different groups are shown in Table 2.

**Figure 1:**
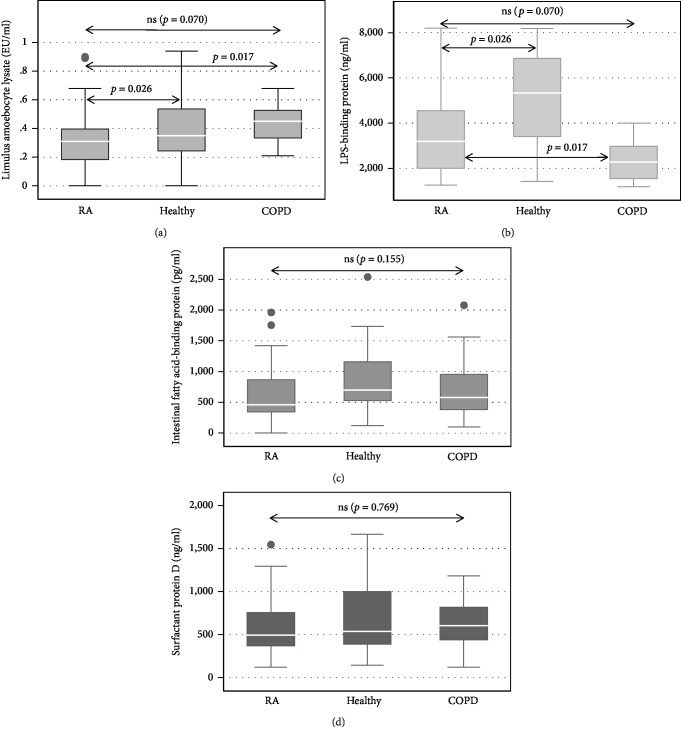
Box and whisker graphs showing median concentrations of the four biomarkers and statistical differences between the groups as per Dunn's post hoc test.

The concentrations of the four test biomarkers of microbial translocation, LPS, SP-D, I-FABP, and LBP for the group of RA patients and both control groups are shown in Figures 1(a)–1(d). Serum concentrations of LPS were significantly lower in the RA group relative to both the healthy (*p* = 0.026) and the COPD (*p* = 0.017) control groups following application of Dunn's post hoc test. Median levels of LBP were significantly different in all groups (*p* = 0.002), as well as between RA and healthy (*p* = 0.036) and RA and COPD patients (*p* = 0.001). With respect to SP-D and I-FABP, serum levels of both of these biomarkers failed to attain statistical significance.

### 3.1. Multiple Pairwise Correlations between the Test Biomarkers, as well as Correlations of These with Indicators of Disease Activity in RA Patients

A weak positive correlation was noted between hs-CRP and LBP (*r* = 0.3812; *p* = 0.0152) in the entire cohort of RA patients, which was strongest in the subgroup of users of tobacco products (*r* = 0.6018; *p* = 0.0137). LBP also showed a trend towards correlating with DAS 28-3 in the subgroup of RA patients that used tobacco products (*r* = 0.4599; *p* = 0.0731), while a moderate correlation between serum SP-D and disease duration (*r* = 0.4181; *p* = 0.0073), as well as weak negative correlations of this biomarker with I-FABP (*r* = −0.2869; *p* = 0.0123) and LPS (*r* = −0.3287; *p* = 0.0411) were also noted.

### 3.2. Multiple Pairwise Correlations of Autoantibodies with Endotoxin, LBP, SP-D, and I-FABP

These results are shown in Table 3. The most notable positive correlations were observed in the subgroup of tobacco users between (i) LPS and RF IgG autoantibodies (*r* = 0.53; *p* = 0.033), (ii) LBP and aCCP IgG autoantibodies (*r* = 0.53; *p* = 0.044), and (iii) LBP and RF IgG antibodies (*r* = 0.51; *p* = 0.04). No statistically significant associations of hs-CRP with either aCCP or RF autoantibodies were detected. Interestingly, a statistically significant negative correlation was detected between SP-D and CCP IgG antibodies in the subgroup of tobacco users (*r* = −0.53; *p* = 0.035). These observations clearly underscore the positive relationships between LPS and elevations in systemic biomarkers of RA.

Wilcoxon's matched-pairs signed-ranks testing of autoantibody isotypes in RA, these being aCCP-IgG and RF-IgM, as included in the ACR/EULAR 2010 scoring criteria for RA diagnosis, revealed that aCCP-IgG and RF-IgM were statistically significantly different (*Z* score = 2.379, *p* = 0.0174), meaning that a highly elevated aCCP-IgG titre in a patient did not necessarily correlate with a highly elevated RF-IgM titre. This finding was consistent across the other isotypes tested (*Z* score range = −3.76–4.28, *p* value range = 0.001–0.042). However, when categorised according to tobacco use, a strong, statistically significant correlation between concentrations of aCCP and RF autoantibodies of the IgG isotype was detected (*r* = 0.72; *p* = 0.0016) in the subgroup of RA tobacco users.

## 4. Discussion

A number of publications have alluded to the possible involvement of the intestinal and pulmonary microbiota, as well as environmental, lifestyle, and occupational factors, in triggering or exacerbating the development of RA in both humans and in murine models of experimental disease [11, 12, 20, 30–36]. However, the potential role of proinflammatory bacterial products, especially endotoxins, reactive with pathogen recognition receptors on cells of the innate immune system, in the immunopathogenesis of RA, remains largely uncertain. This is particularly the case in sub-Saharan Africa, a geographic region where poverty and ongoing urbanisation have been accompanied by an increased prevalence of RA [37], often presenting as severe disease [21–25, 38].

However, in the current study, levels of circulating LPS were found to be significantly lower in the group of RA patients when compared with those of both the healthy and COPD control groups, while levels of I-FABP were comparable between the groups, seemingly excluding leakage of LPS from the GIT. Serum concentrations of the other two biomarkers, SP-D and LBP, were also comparable in all three groups. With respect to associations between isotypes of RF and aCCP autoantibodies in the entire group of RA patients, weak, albeit statistically significant, positive correlations were detected between LPS and aCCP-IgG, as well as between LBP and RF-IgG. LBP also showed a weak, positive, significant correlation with hs-CRP, which is not surprising since both biomarkers function as positive acute phase reactants. A trend towards a positive association between LBP and DAS 28-3 scores was also noted in RA patients exposed to tobacco products, as previously reported by Wen et al. [10]. Although interesting, the slightly lower, albeit statistically significant, levels of circulating LPS observed in the overall group of RA patients relative to the control groups are somewhat surprising and difficult to explain. One possibility, albeit speculative, is that the RA-associated chronic inflammatory response results in accelerated hepatic and/or antibody-mediated clearance of LPS. Alternatively, this discrepancy may result from the lower prevalence of tobacco usage in the RA group relative to the control group in particular.

Despite the apparent lack of associations between circulating LPS and indices of disease activity in the entire group of patients with RA noted in the current study, categorisation of the patients according to tobacco usage proved to be revealing. Statistically significant associations, two novel and one previously described, of smoking with inflammatory biomarkers were identified, further underscoring the well-recognised risks posed by tobacco usage to those with both developing and existing disease. The novel positive associations were those between (i) LPS, as well as its surrogate, LBP, with RF IgG autoantibodies and (ii) LBP with aCCP IgG autoantibodies. The strongly significant positive association between smoking and elevated serum levels of both aCCP and RF autoantibodies detected in the current study has been described previously [39]. A fourth association, a statistically significant negative relationship between serum SP-D and aCCP may seem surprising. However, this may be explained by a finding that smoking, which triggers protein citrullination in the lungs [40, 41], is associated with significantly decreased pulmonary concentrations of SP-D [42].

In the context of the aforementioned findings, it is noteworthy that usage of nasally inhaled snuff, as opposed to cigarette smoking, is common practice among older African females [27], with both types of tobacco usage resulting in similar levels of serum cotinine [43]. Given that tobacco contains high concentrations of LPS [17–19], it is therefore possible that exposure to tobacco-derived bacterial products, as opposed to microbial translocation from the GIT and/or lungs, may account for the associations of LPS/LBP with both types of autoantibodies, as well as for the strong positive interrelationship between aCCP and RF autoantibodies. Moreover, LBP may represent a more accurate indicator of LPS-induced inflammatory stress, possibly due to the instability of, and or complex-forming by, LPS.

## 5. Conclusions

The findings of this study imply that exposure to tobacco products, as opposed to microbial translocation, represents the probable source of LPS in our cohort of RA patients, a contention that is based on the detection of associations, several of which are novel, between cotinine levels, LPS, LBP, and RA-associated autoantibodies.

## Figures and Tables

**Table 1:** Demographics of RA patients.

	*N*	Median	Iqr	Min	Max
Age (Yr)	40	54.5	19	19	79
CDAI	40	23.8	19.8	6	55
DAS 28-3	40	4.8	1.4	2.9	5
SDAI	40	26.4	18.8	7.7	60
hs-CRP	40	12	26.5	1	116
Dur (m)	40	7	5	0	14
RF-IgM	40	46	92,5	1.8	2857
RF-IgA	40	20.0	41.5	4.1	395
aCCP-IgG	40	166	441.5	0.6	1652
aCCP-IgA	40	9.8	33.8	0.9	108

Yr—years; CDAI—Clinical Disease Activity Index; DAS—disease activity score; SDAI—Simplified Disease Activity Index; hs-CRP—high-sensitivity C reactive protein; Dur (m)—disease duration in months; RF—rheumatoid factor; aCCP—anticyclic citrullinated peptides; Ig—immunoglobulin.

**Table 2:** Cotinine levels (ng/ml) of the RA and control groups.

	RA		COPD		Healthy	
	NTU	TU	NTU	TU	NTU	TU
*N*	24	16	6	4	8	12
Median	0	77	0	76	0	95
Iqr	0	94	0	75	0	6
Min	0	5	0	17	0	93
Max	0	133	0	133	0	103

COPD—chronic obstructive pulmonary disease; RA—rheumatoid arthritis; NTU—nontobacco usage; TU—tobacco usage; Iqr—interquartile range.

**Table 3:** Correlations between disease indicators and the various test biomarkers.

		LPS			LBP			SP-D	I-FABP
		All	NTU	TU	All	NTU	TU		
aCCP-IgG	rho	**0.3930**	0.3674	ns	0.2770	ns	**0.5088**	ns	ns
	*p*	**0.0133**	0.0846		0.0836		**0.0442**		
aCCP-IgA	rho	ns			ns		ns		
	*p*								
RF-IgM	rho						0.4731		
	*p*						0.0642		
RF-IgA	rho						ns		
	*p*								
RF-IgG	rho			**0.5324**	**0.3938**	ns	**0.5157**		
	*p*			**0.0334**	**0.0119**		**0.0409**		

Key: aCCP—anticyclic citrullinated peptide; RF—rheumatoid factor; Ig—immunoglobulin; LPS—lipopolysaccharide; LBP—LPS-binding protein; SP-D—surfactant protein D; I-FABP—intestinal fatty acid-binding protein; NTU—nontobacco usage; TU—tobacco usage; ns—not significant.

## Data Availability

The numeric data used to support the findings of this study are available from the corresponding author upon request.
